# What are the global trends in research on resin infiltration in dentistry? An altmetric and bibliometric analysis

**DOI:** 10.1590/1807-3107bor-2025.vol39.067

**Published:** 2025-07-07

**Authors:** Isabela RAMOS, Aurélio de Oliveira ROCHA, Julia Maldonado GARCIA, Luana Hoepers de JESUS, Lucas Menezes dos ANJOS, Pablo Silveira SANTOS, Carla Miranda SANTANA, Mariane CARDOSO

**Affiliations:** (a) Universidade Federal de Santa Catarina – UFSC, Department of Dentistry, Florianópolis, SC, Brazil.

**Keywords:** Dental Caries, Altmetrics, Bibliometrics, Tooth Remineralization, Dentistry

## Abstract

The aim of this study was to analyze the trends and main characteristics of articles on resin infiltration (RI) in dentistry. The search was carried out in August 2023 on Web of Science. Two researchers selected the articles and excluded conference articles. The following data were extracted from the selected articles: citations, year and journal of publication, study design and theme, authorship and institutions, keywords, country and continent. Collaborative networks were generated using the software Vosviewer. Dimensions were consulted to measure altmetric data. Correlation between data was determined by the Sperman’s test. A total of 351 articles were included. The number of citations ranged from 0 to 230. The articles were published between 2007 and 2023. The most prominent journal was Journal of Dentistry (n = 36). The majority were laboratory studies (n = 171) evaluating the performance of RI to mask white spot lesions (n = 248), mainly due to caries (n = 256). The country with the most articles was Brazil (n = 51), however the biggest highlight was Europe (n = 141). The most prominent author was Meyer-Luckel H (n = 33). Vosviewer indicated strong collaborations between authors. According to Dimensions, most citations were from Mendeley followed by X users. This study identified an emerging trend in research on RI in dentistry. Based on this review, most research interest in RI was from Europe, addressing the use of RI to arrest and mask white spot lesions. It is concluded that more intervention studies are needed and that Africa and Oceania have a low publication rate.

## Introduction

Minimal intervention dentistry has been gaining prominence in recent years due to its minimally invasive treatment proposals, which manage the disease process while minimizing the loss of healthy tooth structure.^1^ Nonrestorative or microinvasive treatments are mainly indicated to arrest early-stage caries and to mask the unfavorable tooth discoloration by hypomineralisation of the enamel.^2^

The use of resin infiltration (RI) in microinvasive treatments has been recommended worldwide due to the easy application and high clinical performance.^3,4^ Resin infiltrants are triethylene glycol dimethacrylate (TEGDMA)-based resins that have a low viscosity and a high penetration capacity into the porous enamel lesion by capillary forces^5,6^. It was introduced to conceal the demineralized lesions and maintain the natural translucency of the enamel^7^. RI penetrates hypomineralized enamel lesions, filling and restoring intercrystalline spaces. This creates a polymeric structure that micromechanically binds the enamel prisms, halting the demineralization process and thus inhibiting the progression of caries and masking white spot lesions, as its refractive index is similar to that of dental enamel.^1,8,9^ The high capacity to penetrate the tooth structure has sparked great interest in the use of RI for other dental applications, thus making this material the focus of numerous scientific studies.^4,10^

Resin infiltrants are marketed under the brand name Icon^®^ (DMG, Hamburg, Germany), which is a technology that uses the concept of infiltrating non-cavitated carious lesions with a low-viscosity resin, thereby preventing lesion progression without the need for dental tissue removal.^5^ It comes in two forms: kits for use on proximal or vestibular surfaces.^11^ In this technique, the enamel surface is conditioned with 15% hydrochloric acid for 2 minutes, then the tooth is thoroughly rinsed, after which the low-viscosity resin is injected into the intercrystalline gaps of the demineralized enamel, and the material is light-cured.^7^ RI is used in posterior to remineralize and arrest of white spot lesions, while in anterior teeth, it is also used to mask enamel lesions, ensuring better aesthetic results for the patient.

Bibliometrics uses traditional metrics (scientific citations) to outline the cumulative scientific profile of a given subject.^12^ Bibliometric analysis allows identifying research trends, highlighting topics that have been widely explored or that need more attention from researchers.^13^ Altmetric analysis uses alternative metrics (mentions on online platforms) to identify current interest in research. In 2018, a scientific data platform called Dimensions developed by Digital Science & Research Solutions emerged for altmetric analysis.^11^ Altimetric analysis includes different indicators such as websites, blogs, social networks, and reference manager systmes.^14^ Considering the importance of associated materials to minimal intervention dentistry, metrics-based studies have been published analyzing the scientific status and proposing new avenues to be explored.^15-17^ However, we did not find any reviews analyzing the scientific status of the use of RI in dentistry. Thus, the objective of this study was to analyze the characteristics and performance of research on RI within the scientific environment through bibliometric analysis and in the online environment through altmetric analysis.

## Methods

### Information sources and search strategy

This review was reported in accordance with the Guideline for Reporting Bibliometric Reviews of the Biomedical Literature (BIBLIO).^18^ In August 2023, we conducted an electronic search on Web of Science - Core Collection (WS-CC). We applied the following strategy to retrieve the articles: [TS= (“Resin* Infiltration” OR “Resin* Infiltrant” OR “Infiltrant Resin*” OR “Infiltration Resin*” OR “Icon Resin*” OR “Icon Infiltration” OR “Icon Infiltrant” OR “Icon DMG” OR “Resin-infiltrated” OR “Icon Resin* Infiltration” OR “Icon Resin* Infiltrant”)].

### Eligibility criteria

Two independent researchers (IR and JMG) selected the articles after reading the title, abstract, and/or full text when necessary. Disagreements were resolved by consensus with a third researcher (AOR). Studies associated with the use of RI in dentistry were included. There were no year or language restrictions. Editorial or conference material was excluded.

### Data selection

The following data were collected from the selected articles: number of citations, citation density, year of publication, journal, impact factor (IF) (Journal Citation Reports 2022), study design, theme (type of spot and main objective of the study), country and continent, institution (based on the affiliation of the corresponding author), authors, and keywords.

### Study design and themes

Study design was classified as: laboratory study, interventional study (randomized clinical trial [RCT] or non-randomized clinical trial [NRCT]), observational study, case report, systematic review, and literature review. Regarding the type of lesion investigated, the articles were grouped into “dental caries”, “dental fluorosis”, “enamel hypoplasia”, “molar incisor hypomineralization” and “tooth wear”. Articles that addressed two or more pathologies were classified as “multiple lesions” and articles that did not address the type of lesion were classified as “not identified”.

Regarding main objective, the studies were grouped into: “evaluation of the performance of RI to arrest and mask white spot lesions”, “evaluation of the penetration of RI into the tooth structure”, “evaluation of the tooth structure after application of RI”, “analysis of the properties of RI”, “use of RI to improve the adhesion of orthodontic brackets”, “use of RI to prevent tooth wear (erosion or abrasion)”, and “scientific status” (review articles that investigated the use of RI in dentistry). Studies that evaluated an isolated theme were grouped under “other”.

### Altmetric and bibliometric analysis

Dimensions (dimensions.ai) was used to measure the altmetric data of the article’s performance (performance of publications on social media, traditional media, and online reference managers). The pre-determined search strategy was applied to the Dimensions database without any filters or restrictions on the same day as the search in the WS-CC database. The documents were organized in descending order according to the Altmetric Attention Score (AAS), and all studies with an AAS were analyzed by two independent researchers. The bibliometric collaborative networks were graphically represented using the Visualization of Similarities Viewer software (VOSviewer, version 1.6.17.0, Netherlands), allowing the identification of connections between authors and keywords. In the network analysis, terms corresponding to major foci and sources had higher occurrences. Terms associated with foci of the same color indicate greater collaboration between articles. Lines connecting terms indicate collaboration between them.

### Statistical analysis

We applied the Kolmogorov-Smirnov test to verify the normality of the data that showed a non-normal distribution. Therefore, Spearman’s correlation was applied to determine whether there was a correlation in the number of citations between the year of publication and the journal’s impact factor. For data analysis, we used the statistical software SPSS for Windows (SPSS, version 24.0; IBM, Armonk USA Corp).

## Results

### Search results

**Figure 1:**
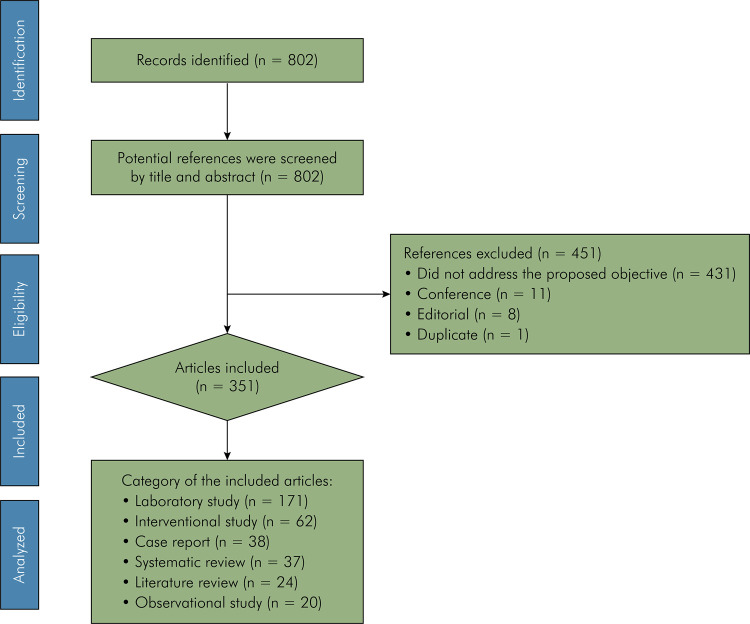
Flowchart of the study selection protocol.

The initial search in WS-CC yielded 802 studies. Of these articles, eight editorial documents, eleven conferences, and one duplicate study were excluded. The remaining articles were excluded because they did not address the proposed objective (n = 431) (Figure 1). Thus, 351 articles were included in this bibliometric analysis.

### Citation analysis

The selected studies had a total of 6,261 citations on WS-CC. Of these, 3,168 were self-citations (50.59%). The study by Frencken JE et al.^19^ with 230 citations, “Minimal intervention dentistry for managing dental caries - a review”, was the most cited article on WS-CC published in the International Dental Journal. The details of the top 10 articles with the highest citations are presented in Table 1. Spearman’s correlation showed a moderate positive correlation between the number of citations and the journal’s impact factor (rho = 0.397) and a strong negative correlation between the number of citations and the year of publication (rho = -0.767). Thirteen studies were cited 100 times of more.

**Table 1:** Top 10 articles in bibliometric performance in citation.

Rank	Article	Citation (WS-CC)
1	BaniHani A, Santamaría RM, Hu S, Maden M, Albadri S. Minimal intervention dentistry for managing carious lesions into dentine in primary teeth: an umbrella review. Eur Arch Paediatr Dent. 2022 Oct;23(5):667-693.	230
2	Paris S, Meyer-Lueckel H, Kielbassa AM. Resin infiltration of natural caries lesions. J Dent Res. 2007 Jul;86(7):662-6.	167
3	Paris S, Hopfenmuller W, Meyer-Lueckel H. Resin infiltration of caries lesions: an efficacy randomized trial. J Dent Res. 2010 Aug;89(8):823-6.	158
4	Kielbassa AM, Muller J, Gernhardt CR. Closing the gap between oral hygiene and minimally invasive dentistry: a review on the resin infiltration technique of incipient (proximal) enamel lesions. Quintessence Int. 2009 Sep;40(8):663-81.	140
5	Meyer-Lueckel H, Paris S. Improved resin infiltration of natural caries lesions. J Dent Res. 2008 Dec;87(12):1112-6.	135
6	Meyer-Lueckel H, Paris S, Kielbassa AM. Surface layer erosion of natural caries lesions with phosphoric and hydrochloric acid gels in preparation for resin infiltration. Caries Res. 2007;41(3):223-30.	135
7	Paris S, Meyer-Lueckel H. Inhibition of caries progression by resin infiltration in situ. Caries Res. 2010;44(1):47-54.	113
8	Paris S, Meyer-Lueckel H, Cölfen H, Kielbassa AM. Resin infiltration of artificial enamel caries lesions with experimental light curing resins. Dent Mater J. 2007 Jul;26(4):582-8.	112
9	Urquhart O, Tampi MP, Pilcher L, Slayton RL, Araujo MWB, Fontana M, Guzmán-Armstrong S, Nascimento MM, Nový BB, Tinanoff N, Weyant RJ, Wolff MS, Young DA, Zero DT, Brignardello-Petersen R, Banfield L, Parikh A, Joshi G, Carrasco-Labra A. Nonrestorative Treatments for Caries: Systematic Review and Network Meta-analysis. J Dent Res. 2019 Jan;98(1):14-26.	110
10	Paris S, Meyer-Lueckel H. Masking of labial enamel white spot lesions by resin infiltration--a clinical report. Quintessence Int. 2009 Oct;40(9):713-8.	109

The article entitled “Best Clinical Practice Guidance for Clinicians Dealing with Children Presenting with Molar-Incisor Hypomineralisation (MIH): An Updated European Academy of Paediatric Dentistry Policy Document” had the highest citation density, with a value of 62. This paper was written by Lygidakis et al.^20^ in 2022 and published in the European Archives of Paediatric Dentistry.

### Year of publication

**Figure 2:**
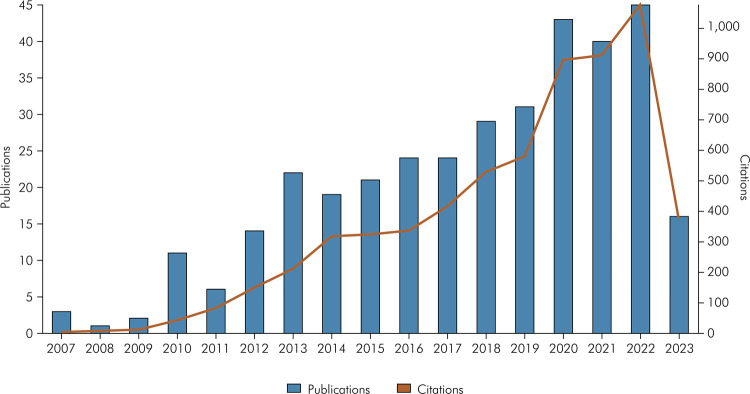
Distribution of the number of publications over the years.

The studies were published between 2007 and 2023, which shows that this topic has been studied in dentistry for 16 years. Published in 2007, three studies were considered the earliest and most pioneering on this subject. The most recent articles were published in 2023 (n = 17). The largest number of articles was published in 2022 (n = 45), demonstrating the current interest in the use of RI in dentistry. A description of the number of publications and citations for each year is shown in Figure 2.

### Journals and Impact Factor

The main journals and impact factors are shown in Table 2. The Journal of Dentistry (n = 36) published most studies related to RI, followed by Operative Dentistry (n = 22) and Caries Research (n = 15). According to Journal Citation Reports, the journals with the highest impact factors (IF) in 2022 linked to this subject were: International Journal of Oral Science (IF 14.9) with one article, ACTA Biomaterialia (IF 9.2) with two articles, and Cochrane Database of Systematic Reviews (IF 8.0) with one article.

**Table 2:** Journals with the most publications on RI.

Journal Title	Number of articles	Impact factor 2022
Journal of Dentistry	36	4,4
Operative Dentistry	22	2,2
Caries Research	15	4,2
Clinical Oral Investigations	13	3,4
Dental Materials Journal	11	2,5
Angle Orthodontist	10	3,4
International Journal of Paediatric Dentistry	9	3,8
Journal of Dental Research	9	7,6
Journal of Esthetic and Restorative Dentistry	9	3,2
Quintessence International	7	1,9

### Study design and themes

Most of the articles were laboratory studies (n = 171), followed by interventional studies (n = 62) (RCT = 39 and NRCT = 23), case reports (n = 38), systematic reviews (n = 37), literature reviews (n = 24), and observational studies (n = 20). Considering themes, the most frequently mentioned lesions were dental caries (n = 256), followed by unidentified lesions (n = 23), fluorosis (n = 21), MIH (n = 21), enamel hypoplasia (n = 18), tooth wear (erosion or abrasion) (n = 6), and multiple lesions (n = 6). Regarding the main objective of the studies, the most prevalent theme was evaluation of the performance of RI to arrest and mask white spot lesions (n = 248), followed by evaluation of the penetration of RI into the tooth structure (n = 31), evaluation of the tooth structure after RI application (n = 18), scientific status (n = 15), use of RI to improve orthodontic bracket adhesion (n = 14), other (n = 11), analysis of RI properties (n = 10), and use of RI to prevent tooth wear (erosion or abrasion) (n = 4).

### Countries and continents

**Figure 3:**
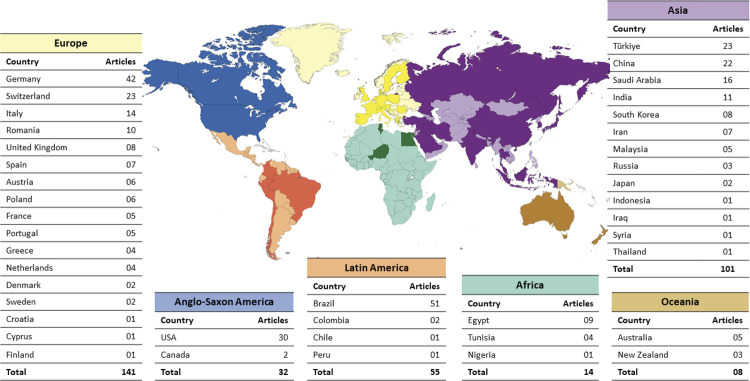
Distribution of country of origin of RI publications.

A total of 42 countries contributed to the articles related to RI. The three countries with the highest number of publications were: Brazil (n = 51), Germany (n = 42), and the United States of America (USA) (n = 30). Among the continents with the most articles (shown in Figure 3), Europe stands out (n = 141), followed by Asia (n = 104) and Latin America (n = 55).

### Institutions

A total of 195 institutions contributed to the articles related to RI. Table 3 shows the institutions with the highest number of publications. With 11 publications each, the top three positions were University of Bern (Switzerland), University of Kiel (Germany), and the State University of São Paulo (Brazil).

**Table 3:** Main institutions associated with RI research.

Institution	Country	Number of articles
University of Bern	Switzerland	11
University of Kiel	Germany	11
State University of São Paulo	Brazil	11
Charite University Medicine Berlin	Germany	10
University of Zurich	Switzerland	9
RWTH Aachen University	Germany	8
University of São Paulo	Brazil	7
Danube Private University	Austria	6
Mansoura University	Egypt	6
King Abdulaziz University	Saudi Arabia	5
University of Ege	Turkey	5
Campinas State University	Brazil	5

### Contributing authors

**Figure 4:**
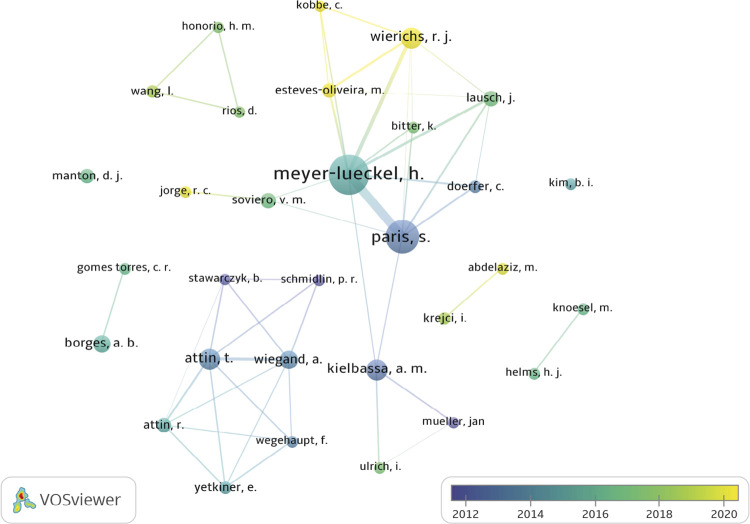
Main groups/authors that have researched RI. Names written in a larger font and associated with the largest points are the most frequent authors. Authors associated with blue points are related to older studies and those with yellow points are related to more recent studies, as the scale indicates.

A total of 1,376 authors participated in the selected articles. Table 4 shows the authors with the highest number of publications. With 33 articles, Meyer-Luckel H was the author with the most publications, followed by Paris S with 25 articles, Attin T, Wierichs RJ, and Kielbassa AM with 11 articles each. The frequency (5 or more occurrences) of their appearance and the co-authorship relationship between them is shown in Figure 4.

**Table 4:** Authors with the highest number of publications on RI.

Authors	Number of articles	Number of citations
Meyer-Lueckel H	33	1,597
Paris S	25	1,538
Kielbassa AM	11	693
Attin T	11	183
Wierichs RJ	11	86
Wiegand A	9	171
Borges AB	8	266
Lausch J	7	138
Soviero VM	7	99
Manton DJ	6	303
Attin R	6	72
Esteves-Oliveira M	6	42

### Keywords

**Figure 5:**
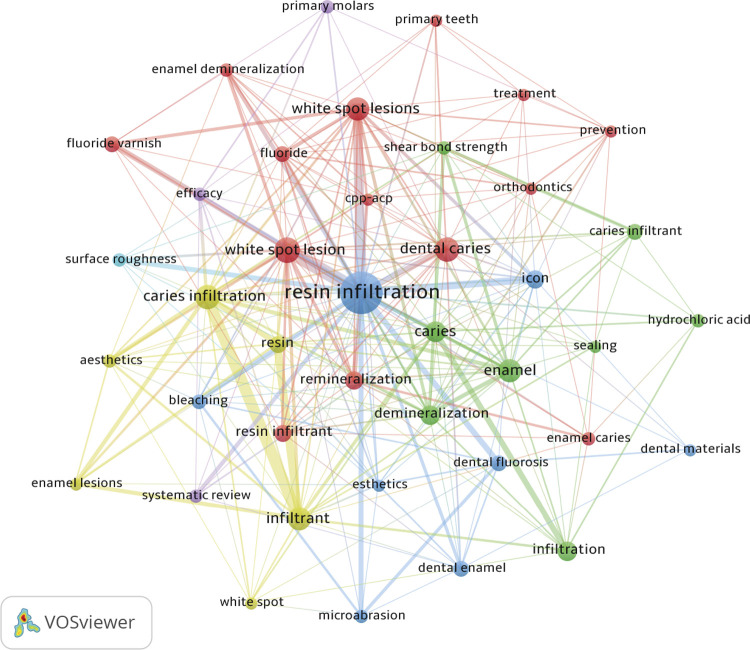
Frequency and interaction of the main keywords associated with the study. Terms associated with larger points and written in a larger font appeared more often. The lines joining the points indicate the relationship and use of these words in the same studies. Points with the same color indicate greater collaboration between the terms.

We identified a total of 626 keywords. The most prevalent was “resin infiltration” (100 occurrences), followed by “white spot lesion” (32 occurrences) and “dental caries” (29 occurrences). Figure 5 shows the most prevalent keywords (6 or more occurrences) and the collaborative relationships between them.

### Altmetric analysis

According to Dimensions, 65 articles from RI in dentistry have metrics for mentions on social media (Facebook, Twitter, blogs, and YouTube), traditional media (general or scientific sites), online reference managers (Mendeley), and patents. The article with the highest altmetric performance was “Micro-invasive interventions for managing proximal dental decay in primary and permanent teeth”^21^ with an AAS of 89, which was mentioned by one news outlets, 6 blogs, 56 X users, 5 Facebook pages, 2 Wikipedia pages, 1 Google+ posts, and 529 Mendeley entries. The details of the top 10 articles with the highest AAS are presented in Table 5.

**Table 5:** Top 10 articles with the highest altmetric performance.

Altmetric Attention Score	Article	Mentioned by
**89**	Dorri M, Dunne SM, Walsh T, Schwendicke F. Micro-invasive interventions for managing proximal dental decay in primary and permanent teeth. Cochrane Database Syst Rev. 2015 Nov 5;2015(11):CD010431.	**1** news outlet
		**6** Blogs
		**56** X users
		**5** Facebook pages
		**2** Wikipedia
		**1** Google+ Posts
		**529** Mendeley
**69**	Slayton RL, Urquhart O, Araujo MWB, Fontana M, Guzmán-Armstrong S, Nascimento MM, Nový BB, Tinanoff N, Weyant RJ, Wolff MS, Young DA, Zero DT, Tampi MP, Pilcher L, Banfield L, Carrasco-Labra A. Evidence-based clinical practice guideline on nonrestorative treatments for carious lesions: A report from the American Dental Association. J Am Dent Assoc. 2018 Oct;149(10):837-849.e19.	**4** News Outlets
		**1** Blogs
		**31** X Users
		**1** Facebook pages
		**419** Mendeley
**34**	Urquhart O, Tampi MP, Pilcher L, Slayton RL, Araujo MWB, Fontana M, Guzmán-Armstrong S, Nascimento MM, Nový BB, Tinanoff N, Weyant RJ, Wolff MS, Young DA, Zero DT, Brignardello-Petersen R, Banfield L, Parikh A, Joshi G, Carrasco-Labra A. Nonrestorative Treatments for Caries: Systematic Review and Network Meta-analysis. J Dent Res. 2019 Jan;98(1):14-26.	**1** News outlets
		**1** Blogs
		**20** X users
		**502** Mendeley
**21**	Cocco AR, Lund RG, Torre E, Martos J. Treatment of Fluorosis Spots Using a Resin Infiltration Technique: 14-month Follow-up. Oper Dent. 2016 Jul-Aug;41(4):357-62.	**3** News outlets
		**85** Mendeley
**15**	Shivanna V, Shivakumar B. Novel treatment of white spot lesions: A report of two cases. J Conserv Dent. 2011 Oct;14(4):423-6.	**1** News outlets
		**1** Blogs
		**8** Facebook pages
		**52** Mendeley
**15**	Lagarde M, Vennat E, Attal JP, Dursun E. Strategies to optimize bonding of adhesive materials to molar-incisor hypomineralization-affected enamel: A systematic review. Int J Paediatr Dent. 2020 Jul;30(4):405-420.	**1** News outlets
		**1** Blogs
		**1** X users
		**168** Mendeley
**14**	Cazzolla AP, De Franco AR, Lacaita M, Lacarbonara V. Efficacy of 4-year treatment of icon infiltration resin on postorthodontic white spot lesions. BMJ Case Rep. 2018 Jul 18;2018:bcr2018225639.	**1** News outlets
		**1** X users
		**1** Patent
		**112** Mendeley
**14**	Höchli D, Hersberger-Zurfluh M, Papageorgiou SN, Eliades T. Interventions for orthodontically induced white spot lesions: a systematic review and meta-analysis. Eur J Orthod. 2017 Apr 1;39(2):122-133.	**2** Blogs
		**3** X users
		**272** Mendeley
**13**	Doméjean S, Ducamp R, Léger S, Holmgren C. Resin infiltration of non-cavitated caries lesions: a systematic review. Med Princ Pract. 2015;24(3):216-21.	**1** News outlets
		**4** X users
		**264** Mendeley
**12**	Meyer-Lueckel H, Balbach A, Schikowsky C, Bitter K, Paris S. Pragmatic RCT on the Efficacy of Proximal Caries Infiltration. J Dent Res. 2016 May;95(5):531-6.	**1** News outlets
		**3** X users
		**144** Mendeley

## Discussion

Minimally invasive dentistry is characterized by the adaptation of techniques and use of materials in order to increase the preservation of healthy tooth structure, thus reducing unnecessary tooth wear.^17^ RI has been used according to the theory of minimally invasive dentistry, making this material a major focus for use in clinical practice and scientific research.^3^ Therefore, the present study aimed to analyze the main characteristics of the articles related to the use of RI in dentistry. Primarily, most of the studies originate from the European continent and were usually laboratory-based studies investigating the performance of RI in arresting and masking white spot carious lesions.

The main objective of minimally invasive dentistry is to prevent damage the dental tissue as much as possible.^17^ In this scenario, there are non-invasive or micro-invasive procedures where no or only a micrometric portion of dental structure is removed.^1,22^ RI is considered a micro-invasive procedure, as it requires prior acid conditioning and the removal of a micrometric superficial layer of dental structure without resulting in cavitation of the tissue.^1,7^ The present study showed that these terminologies are often erroneously used as synonyms. Therefore, it is suggested that future studies standardize the terminology, considering the use of RI as a micro-invasive treatment.

In clinical practice, dentists are increasingly being sought to treat tooth discoloration, including enamel opacities, which occur due to the loss of the mineral phase of enamel and its replacement by organic fluids, resulting in a chemical change in the substrate’s composition.^23^ These changes present as white spots and are the result of post-eruptive damage, such as dental caries, or pre-eruptive conditions such as dental fluorosis, traumatic hypomineralization, and molar incisor hypomineralization.^23^ These lesions can be associated with dental hypersensitivity and affect esthetics, causing discomfort to patients.^11^ Treating white lesions with the RI technique has several advantages, including preserving tooth structure, halting the progression of initial carious lesions, eliminating the risk of postoperative sensitivity and pulpal inflammation, and improving esthetic outcomes when used to mask white spots.^11^ It is a safe and efficient treatment option for buccal surfaces, which have an important esthetic role. Besides that, the effect of RI is beneficial both immediately after application and in the long term.^7^

The article with the highest number of citations was entitled “Minimal intervention dentistry for managing dental caries - a review”, with a total of 230 citations.^19^ This article discusses and describes the history of minimal intervention dentistry for the management of dental caries and several devices that can be used for detection of carious lesions, prevention measures, and regular and minimally invasive restorative therapies. Among the therapies described, the study outlines the importance and use of RI in minimal intervention dentistry. As a review article that brought together several evidence-based protocols and restorative materials, the study is useful in several types of research, and this may justify its prominence regarding the high number of citations. Other bibliometric studies have also found review articles among the most cited articles on a given subject.^24-26^Papers cited more than 100 times can be considered literature classics.^27^ In this study, only thirteen classics were identified, which may be attributed to the fact that RI is a specific topic with a relatively recent research trajectory.

The scientific evidence in the RI literature is mostly based on laboratory studies (49%). There was further popularization of the use of RI with the creation and widespread clinical use of the Icon resin (DMG, Hamburg, Germany).^28^ This fact may justify the massive amount of laboratory testing of this material to arrest and mask white spot lesions, its relationship with tooth structure, and its use in many other dental applications.^4,10^ However, a higher level of scientific evidence is mainly linked to high-quality studies, such as randomized controlled trials and systematic reviews. Reliable evidence from well-conducted clinical trials and systematic reviews plays a key role in evidence-based dentistry.^15^ We observed a relatively low number of clinical trials (n=62) and systematic reviews (n=37) in this study. Almost half of the clinical trials were non-randomized. This may be related to the difficulty of standardizing white spot lesions to ensure a robust sample for clinical trials.^29^ In addition, the low number of RCTs may be related to the high level of detail required to design RCTs, as well as the high cost of materials, conducting clinical follow-up over time, and other limitations related to the statistical inference and low internal or external validity.^30^ Well-conducted RCTs are considered the gold standard for clinical decision in evidence-based dentistry.^27^ Therefore, there is a clear need for more studies with robust and reliable methodology on the use of RI in dentistry.

RI has been found to be of great interest in dental caries. Untreated dental caries affected 2.3 billion people worldwide in 2017, making it the most common health condition in the global population.^31^ Literature data show that the prevalence of white spot carious lesions in primary teeth reaches 14%, and this value increases for children in low-income settings.^32^ In permanent teeth in patients undergoing orthodontic treatment, the prevalence of patients with one or more carious lesions is 79.3%.^33^ The high worldwide prevalence of initial dental caries may justify the noticeable trend of publications on RI for the clinical control of this condition. This also explains why the main objective of the articles was the use RI to arrest and mask white spot lesions.

With the growing esthetic appeal, there has been a greater demand for restoring lesions caused by defects in the quality and quantity of enamel (fluorosis, hypoplasia, MIH).^34^ However, enamel wear can weaken the tooth. Based on the precepts of minimal intervention, there has been a strong trend towards investigating the use of RI to mask these lesions.^35^ Another interesting trend is the use of RI prior to bonding orthodontic brackets, as the penetration of RI into the dental structure could lower the rate of adhesion failure of these pieces to the teeth.^10^

In developing countries, the reduction in dental caries is delayed, with still a high prevalence rate.^36^ This may explain the predominance of articles originating from Brazil. Despite language barriers, gaps in professional networks, and limited access to information, Brazil has stood out in other bibliometric analyses.^13^ The state of Sao Paulo plays a very important role in the high number of publications because of the University of Sao Paulo. This university ranks first in Brazil and 85th in the world according to the QS World University Ranking 2024.^37^ It also has an excellent infrastructure and financial resources to support significant research endeavors.^26^ However, Germany also stood out. This may be related to the fact that the most popular RI (ICON) is from that country.

Despite Brazil’s prominence, the continent with the highest number of publications was Europe, since it has the greatest number of countries (n=17). The same result was observed in another bibliometric analysis.^24^ However, the present study highlighted the need for scientific collaboration with publications on RI in Africa and Oceania. The prominence of Brazil and Germany corroborates the origin of the most frequent institutions, which were the University of Kiel (Germany) and the State University of São Paulo (Brazil). However, the University of Bern (Switzerland) was equally relevant.

Among the most prominent authors, we observed that Meyer-Luckel, Paris S, Wierichs RJ, and Kielbassa AM participated in the same research group and, together, they carried out mostly laboratory and interventional studies to investigate the efficacy and penetration of RI to arrest and mask caries white spot lesions. However, Attin T, who also had an important number of studies despite not participating in the aforementioned group, also investigated mainly the efficacy and penetration of RI to arrest and mask caries white spot lesions through laboratory studies. Figure 4 shows the collaboration network among authors and shows a limited number of prominent groups involved in research on RI. This may explain the high percentage of self-citation (50.59%), as it is a recent topic with few researchers investigating this technique. Another study found similar rates of self-citation^38^. Self-citation might be linked to the continuous development of the RI topic, allowing older works to support ongoing research and the introduction of new data.

Digital Science & Research Solutions launched “Dimensions” in 2018, an online academic platform that combines and displays traditional metrics (scientific citations) and alternative metrics (online mentions) providing an immediate overview of the interest of researchers and professionals through data altmetrics.^39^ The platform takes into account several indicators such as awards, publications in journals and books, mentions on social media, academic citations, clinical trials and commercial patents.^14^ According to altmetric data, the most popular article on RI in dentistry was a systematic review that aimed to evaluate the effects of microinvasive treatments in the management of proximal caries lesions in the primary and permanent dentition in children and adults.^21^ This study had significant interest among Mendeley and X users and achieved an AAS of 89. Only one study showed high interest in traditional and alternative metrics, ranking among the first 10 in each category.^2^It is important to highlight that, altmetric analysis shows the panorama of studies on internet and social media and differs from bibliometric analysis, which assessed scientific production in the academic environment such as scientific databases.^39^ Bibliometrics require more time to reflect research interest, as it is related to citations, while altimetric data indicate an immediate interest in a particular topic and complement the bibliometric analysis.^40^ This difference explains why some top cited articles do not appear in the AAS results, which has been found in other studies.^39^

The strength of this bibliometric and altmetric study lies in the fact that no filters were applied to restrict year of publication, number of citations, or language, allowing for a comprehensive global analysis of the articles published on this topic up to the date of the search. However, a limitation is that only one database was used to extract the selected articles, although it is considered the most prestigious for bibliometric surveys.^24^ Additionally, there are inherent risks in the data selection and interpretation processes. To mitigate this, two researchers with experience in bibliometric reviews conducted the study and cross-checked all collected data.

## Conclusion

This bibliometric review shows the current state and trends of RI-related research, indicating that currently there is greater research interest in Europe. Meyer-Luckel H was the author with the most publications. There has been a growing number of studies published on RI over the years, reflecting the increasing research interest in this field. Most of the articles were published in the Journal of Dentistry, and most of them were laboratory studies on the application of RI to arrest and mask white spot lesions, especially carious lesions. The low number of publications from Africa and Oceania is a factor that have been little explored. Furthermore, specific intervention studies could ensure the scientific development of this topic. Altmetric analysis revealed that Mendeley’s users had the greatest interest in this topic, followed by X users demonstrating the current interest in this topic in dentistry.

## Data Availability

The contents underlying the research text are contained in the manuscript.
